# Pharmacokinetic/pharma-codynamic study of pralurbactam (FL058) combined with meropenem in a neutropenic murine thigh infection model

**DOI:** 10.3389/fmicb.2024.1516979

**Published:** 2024-12-17

**Authors:** Zhiwei Huang, Wenfang Li, Ruohao Zhang, Yi Li, Xin Li, Xingchen Bian, Shansong Zheng, Xinmei Wang, Ning Zhang, Cong Gao, Beining Guo, Zhenling Wang, Jing Zhang, Xiaojie Wu

**Affiliations:** ^1^Institute of Antibiotics, Huashan Hospital, Fudan University, Shanghai, China; ^2^State Key Laboratory of Biotherapy and Cancer Center, West China Hospital, Sichuan University, Chengdu, Sichuan, China; ^3^Clinical Pharmacology Research Center, Huashan Hospital, Fudan University, Shanghai, China; ^4^Qilu Pharmaceutical Co., Ltd., Jinan, Shandong, China

**Keywords:** pralurbactam, β-lactamase inhibitor, *Klebsiella pneumoniae*, *Escherichia coli*, pharmacokinetic/pharmacodynamic analysis, neutropenic thigh infection model

## Abstract

**Introduction:**

Pralurbactam (FL058) is a novel β-lactamase inhibitor with good inhibitory activity on class A, C, and D β-lactamases. This study aimed to evaluate the pharmacokinetic/pharmacodynamic (PK/PD) relationship of pralurbactam/meropenem in a neutropenic murine thigh infection model.

**Methods:**

After 2-h infection, neutropenic mice was treated with meropenem every 2 h alone or in combination with pralurbactam at different dosing frequencies for 24 h, and the colony count in the thighs was determined before and after treatment. The maximum effect model was fit to the PK/PD relationship to determine the PK/PD index and targets for pralurbactam in combination with meropenem resulting in a static effect and 1-log_10_ kill.

**Results:**

The plasma drug concentration-time data demonstrated that the PK profiles of pralurbactam were consistent with a one-compartment model. Pralurbactam demonstrated a linear PK profile in mice plasma. The percent time of free drug above 1 mg/L (%*f*T > 1 mg/L) was the PK/PD index that best described the bacterial killing effect of pralurbactam/meropenem over 24 h. When the PK/PD index %*f*T > 1 mg/L reached 38.4% and 63.6%, pralurbactam/meropenem combination would achieve bacteriostatic effect and 1-log_10_ reduction against *Klebsiella pneumoniae* in thigh bioburden, respectively.

**Conclusion:**

These PK/PD data derived from mouse thigh infection models will be used to inform the optimal dosing regimen of pralurbactam/meropenem combination in clinical trials.

## 1 Introduction

The clinical use of antimicrobial agents has achieved great success in the treatment of infectious diseases. However, inappropriate use and even abuse of antimicrobial agents have imposed increasing pressure on the pathogenic microorganisms and induce the emergence of antimicrobial resistance due to various mechanisms, which allows the antibiotic-resistant bacteria to escape from killing and so leads to the failure of antimicrobial treatment. One important mechanism of antimicrobial resistance is the production of β-lactamases, which can hydrolyze β-lactam (BL) antibiotics directly. The evolvement of β-lactamases has directly led to the increasing resistance and spread of multidrug-resistant bacteria because of structural variations of the emerging enzymes. According to the results of Antimicrobial Resistance Surveillance System in China (CHINET) (Guo et al., 2022), about 73% of the clinical isolates collected from hospitals across China in 2021 were Gram-negative bacteria, about 60% of which were Enterobacterales. The prevalent β-lactamases-producing Enterobacterales (especially *Klebsiella pneumoniae*) are difficult to treat in clinical practice because they are resistant to nearly all BLs, including carbapenems such as meropenem and imipenem. The commonly used β-lactamase inhibitors (BLIs) such as clavulanic acid, tazobactam, and sulbactam are effective only against class A and D enzymes but not so active on the emerging carbapenemases such as KPC and NDM (Wang et al., 2018). Several novel BL/BLI combinations have been developed in clinical trials to address the issue of carbapenemase-producing Enterobacterales strains (Drawz et al., 2014).

Pralurbactam (FL058) is a novel BLI with a diazabicyclo structure similar to avibactam that is active against class A, C, and D β-lactamases *in vitro* (Sharma et al., 2016). Results of the *in vitro* study showed that pralurbactam in combination with meropenem might be a potential treatment for KPC- and/or OXA-48-producing Enterobacterales infection (Huang et al., 2024a). An *in vitro* susceptibility study (to be published) demonstrated that pralurbactam alone exhibited notable inhibitory effects on *Escherichia coli* and meropenem combined with 4 μg/ml pralurbactam had significantly lower the minimum inhibitory concentration (MIC) for NDM-producing *E. coli* with the MIC_90_ of 0.5 mg/L and exhibited partial inhibitory activity against NDM-producing *K. pneumoniae* with MIC_50_ and MIC_90_ values of 0.25 and 4 mg/L, respectively. Moreover, a completed phase I clinical trial showed that pralurbactam exhibited good safety, tolerance, and PK profiles (Huang et al., 2024b).

Pharmacokinetic/pharmacodynamic (PK/PD) models are usually used to simulate the PK process of antibiotics in human body due to the flexibility and convenience in experimental design (Drawz et al., 2014). The *in vitro* PD data combined with the *in vivo* PK data of different pralurbactam/meropenem combinations can be used to profile the free threshold concentration (C_*T*_) of pralurbactam, PK/PD index, and the target values of pralurbactam/meropenem combinations in animal infection models. In this study, we explored the PK/PD profile of pralurbactam/meropenem combinations in the neutropenic murine thigh infection models caused by different β-lactamases-producing Enterobacterales strains. The PK/PD index and target value for predicting the efficacy of pralurbactam/meropenem obtained from this study will be used to support the dose selection and breakpoint setting in clinical trials (Huang et al., 2024b).

## 2 Materials and methods

### 2.1 Compounds

Pralurbactam (batch nos.: B0220E01 and B0220E01K) was provided by Qilu Pharmaceutical Co. Ltd. Meropenem (batch no.: 2416C) was obtained from Japan Sumitomo Pharmaceutical Co. Cyclophosphamide (CTX, batch no.: 19111925) was provided by Jiangsu Hengrui Medicine Co., Ltd.

### 2.2 Strains

The study used a total of six strains of KPC-, NDM-, or OXA-producing Enterobacterales (three strains of *K. pneumoniae* and four strains of *E. coli*), which were provided by Microbiology Division, Institute of Antibiotics, Huashan Hospital, Fudan University. Quality control strains are selected for each bacteria. The MICs of pralurbactam, meropenem, and pralurbactam/meropenem combination against the strains are shown in Supplementary Table 1.

### 2.3 Neutropenic murine thigh infection models

Mice (SPF grade, female, 25–30 g, 6–7 weeks) were provided by Beijing Huafukang Biotechnology Co. The animal study was reviewed and approved by the Animal Ethics Committee of State Key Laboratory of Biotherapy, Sichuan University (approval letter No.: 20190923035). All experimental animals from the study were intraperitoneally injected with cyclophosphamide (solution using 0.9% sodium chloride injection to achieve a concentration of 15 mg/ml) at a dose of 150 mg/kg at 4 days and 1 day prior to the day of infection to form immunocompromised mouse models. The hair on both thighs of the mice was shaved off 2 h before inoculation. The injection site was disinfected with 75% alcohol before intramuscular injection and dried with skimmed cotton. The mouse was challenged by inoculum of 1 × 10^8^ CFU/ml of the test strain via injection of 50 μl of bacterial solution into both thighs. The time was referred to as *t* = 0 h, 2 h after the mice were infected. The control mice were euthanized at this time to record the initial bacterial colonies by taking the thigh muscle. The specific dosage of pralurbactam or meropenem for each dosing regimen was intraperitoneally injected (*t* = 0 h). At 26 h after the mice were infected (*t* = 24 h), the mice (experimental group) in each dosage cohort were euthanized to record the bacterial load to calculate CFU change (Δlog_10_ CFU).

### 2.4 Quantification

The ultra-high performance liquid chromatography-tandem mass spectrometry (UPLC-MS/MS) system consisted of an LC-30A UPLC (Shimadzu Corporation, Japan) equipped with an Atlantis^®^ T3 (3 μm, 2.1 × 100 mm) column, liquidity, and the cleaning solution mixed with formic acid, ammonium acetate, and acetonitrile, an API5500 mass spectrometer (AB SCIEX, USA). The retention times of chromatographic peaks and mass-to-charge ratios were obtained by MRM scanning pralurbactam (m/z 383.2 → 303.0), meropenem (m/z 384.1 → 141.2), internal standards pralurbactam-D4 (m/z 387.3 → 307.1), and meropenem-D6 (m/z 390.1 → 147.2) for peak identification. The drug concentration in the samples was calculated using Watson LIMS (version 7.5).

The UPLC-MS/MS method used to measure pralurbactam and meropenem concentrations in mice plasma was validated accurately in terms of selectivity, interaction, matrix effect, calibration curve, linearity, precision, accuracy, recovery, and stability. Briefly, the lower limit of quantification (LLOQ) was 0.125 mg/L for pralurbactam and meropenem. At this LLOQ level, the intra- and inter-assay precision was 3.5%–9.0% and 6.3% for pralurbactam, 5.0%–10.7% and 14.3% for meropenem. The overall recovery of pralurbactam and meropenem was 81.2% and 58.6%, respectively, and the corresponding coefficient of variation (CV) was 8.0% and 5.4%. This assay method was applied to quantify pralurbactam and meropenem for PK study.

### 2.5 PK studies

The administration method for pralurbactam or meropenem (dissolved in sterile water) is single-dose intraperitoneal injection. Mice were injected intraperitoneally with pralurbactam alone (5, 50, and 500 mg/kg), meropenem alone (50 and 100 mg/kg) and pralurbactam/meropenem combination (50/100 and 250/100 mg/kg). Five mice were randomly selected for anesthesia and orbital blood sampling at each time-point (0.083, 0.25, 0.5, 1, 2, 3, 4, and 6 h) after dosing. A blood sample (600–800 μl) was obtained from the mouse immediately. Each mouse selected for blood sampling at each time point was euthanized immediately. The concentrations of pralurbactam and meropenem in plasma samples were determined using the validated UPLC-MS/MS method. PK parameters (including non-compartmental parameter linear fitting and compartmental simulation) of pralurbactam and meropenem in plasma were calculated using Phoenix WinNonlin (version 8.1, Certara Corporation). The compartmental PK parameters of pralurbactam were used to calculate the *f*C_max_, *f*AUC_0–24_, and %*f*T > C_T_. The free fraction of pralurbactam was 100% based on *in vitro* mouse plasma.

### 2.6 Pralurbactam combined with meropenem in PD studies

Different dosing regimens will be evaluated in established mouse thigh infection models to assess their effectiveness in killing bacteria, using the change in bacterial count in the mice thigh over 0–24 h post-administration. Five mice will be used for each dosing regimen in *in vivo* model to reduce intra-group variability.

The dose-fractionation experiment was performed in the infection models caused 1 strains of *K. pneumoniae* (17-R1-16), where the dose of meropenem was fixed in 100 mg/kg and the dosing interval was q2h. The dosing interval of pralurbactam changed at q2h, q4h, and q8h, where the daily dose of pralurbactam increased at 0–2,400 mg/kg as specified in the treatment protocol. The dose-escalation experiment was investigated in the infection models established by two strains of *K. pneumoniae* and four strains of *E. coli*, where the dose of meropenem was fixed in 100 mg/kg for *K. pneumoniae* and 50 mg/kg for *E. coli* and the dosing interval was q2h. The dosing interval of pralurbactam was fixed in q2h, where the dose of pralurbactam increased at 0, 5, 10, 25, and 100 mg/kg as specified in the treatment protocol (different in *K. pneumoniae* and *E. coli*). The dosing regimens are shown in Supplementary Table 2.

### 2.7 PK/PD analyses

The correlation between the PK/PD index (%*f*T > C_T_, *f*AUC, *f*C_max_) and the bactericidal effect (Δlog_10_CFU) of pralurbactam in the presence of fixed dose of meropenem and different dosing regimens was fitted separately by employing a non-linear sigmoidal maximum-effect (E_max_) model in Equation 1 (Phoenix WinNonlin, version 8.1, Certara). The value of PK/PD index corresponding to stasis (Δlog_10_CFU = 0) and 1-log10 kill (Δlog_10_CFU = 1) effects was derived for pralurbactam.


(1) $$E=E_{0}-\frac{E_{m\,a\,x}\cdot X^{\gamma }}{E\,C_{50}^{\gamma }+X^{\gamma }}$$

*E* was the pharmacodynamic index (ΔLog_10_CFU). *E*_0_ was the baseline value. *E*_max_ was the maximum bactericidal effect *in vivo*. X was the PK/PD index value *in vivo*. EC_50_ was the PK/PD index value when the bactericidal effect was 50%. γ was the slope of the curve.

## 3 Results

### 3.1 PK profiles of pralurbactam

**FIGURE 1:**
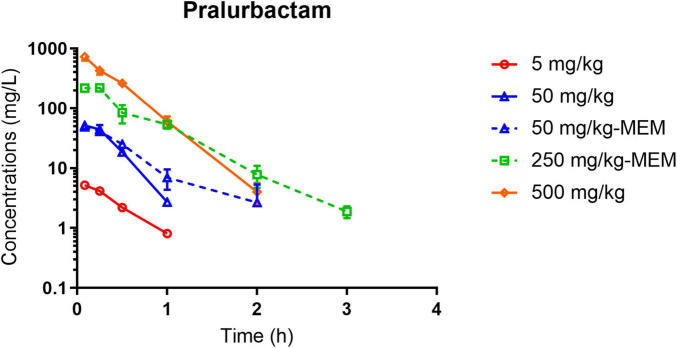
Pralurbactam plasma concentration-time curves after intraperitoneal injection of single ascending doses of pralurbactam or combined with meropenem in mouse thigh infection models. Solid lines indicate pralurbactam alone and dashed lines denote pralurbactam combined with meropenem. The dose of meropenem (combined with pralurbactam) was fixed at 100 mg/kg. MEM, meropenem.

The plasma concentration-time curves after intraperitoneal injection of single dose of pralurbactam alone, and pralurbactam/meropenem are shown in Figure 1. The regression analysis of PK parameters based on non-compartmental models confirmed that the C_max_ or AUC_inf_ of pralurbactam were dose proportional according to concentration data. The linear fitting equation of pralurbactam C_max_ and AUC_inf_ to dose was *y* = 1.049*x* = 0.118 (weighed *R*^2^ = 0.992), and *y* = 1.077*x* = 0.876 (weighed *R*^2^ = 0.999), respectively. According to different pralurbactam or meropenem concentrations in mice plasma obtained using different dosing regimens, the pharmacokinetic process of pralurbactam and meropenem in mice could be described by one-compartment model (PK parameters in Table 1).

**TABLE 1:** Pharmacokinetic parameters of pralurbactam in mice plasma estimated by the one-compartment model.

Compound	Dose	Combined with	K_a_ (h^1^)	K_e_ (h^1^)	V/F (L)
Pralurbactam	5 mg/kg	None	27.4	2.24	0.77
	50 mg/kg	None	13.7	3.83	0.55
	50 mg/kg	Meropenem	24.5	2.11	0.79
	250 mg/kg	Meropenem	26.0	1.93	0.91
	500 mg/kg	None	159.4	2.64	0.57
	Mean		50.2	2.55	0.72

K_a_, first-order absorption rate constant; K_e_, first-order elimination rate constant; V/F, apparent volume of distribution, where F is the bioavailability.

### 3.2 PD results of pralurbactam/meropenem in murine thigh infection models

**FIGURE 2:**
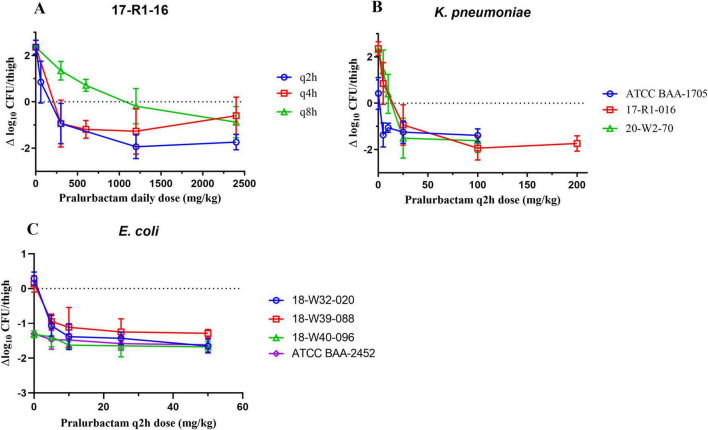
Bactericidal effect of pralurbactam combined with meropenem in terms of pralurbactam daily dose and dose fractionation **(A)**, and caused by *Klebsiella pneumoniae* **(B)** or *Escherichia coli* **(C)** after increasing doses of pralurbactam. The dose of meropenem was fixed in 100 mg/kg for *K. pneumoniae* and 50 mg/kg for *E. coli*. The dosing interval was q2h.

The neutropenic murine thigh infection models were established using the strains of *K. pneumoniae* and *E. coli* that produce different types of carbapenemase (KPC, OXA, or NDM). The bacterial killing curves for different dosing regimens were plotted based on the bacterial counts obtained at various time points (Figure 2). The change in bacteriality from pre- and 24 h after dosing was used as the pharmacodynamic evaluation indicator. PK/PD relationship of pralurbactam/meropenem combination was investigated in the models by dose fractionation (Figure 2A) and increasing the dose of pralurbactam in the combinations (Figures 2B, C). The bactericidal effect of pralurbactam/meropenem combination was related to the daily dose and dosing frequency of pralurbactam when fixed dosing of meropenem. Shorter dosing interval or higher dose of pralurbactam could boost the bacteriostatic effect of each treatment. The combination of at least 25 mg/kg pralurbactam (fixed 100 mg/kg q2h of meropenem) could inhibit the growth of all the test strains of *K. pneumoniae* (17-R1-16, 20-W2-70, and ATCC BAA-1705) more effectively. It is surprising that dosing 50 mg/kg meropenem alone (without pralurbactam) exhibits significant growth inhibition on two strains of *E. coli* (18-W40-096 and ATCC BAA-2452). The two strains of *E. coli* were excluded PK/PD analyses.

### 3.3 PK/PD analyses

**FIGURE 3:**
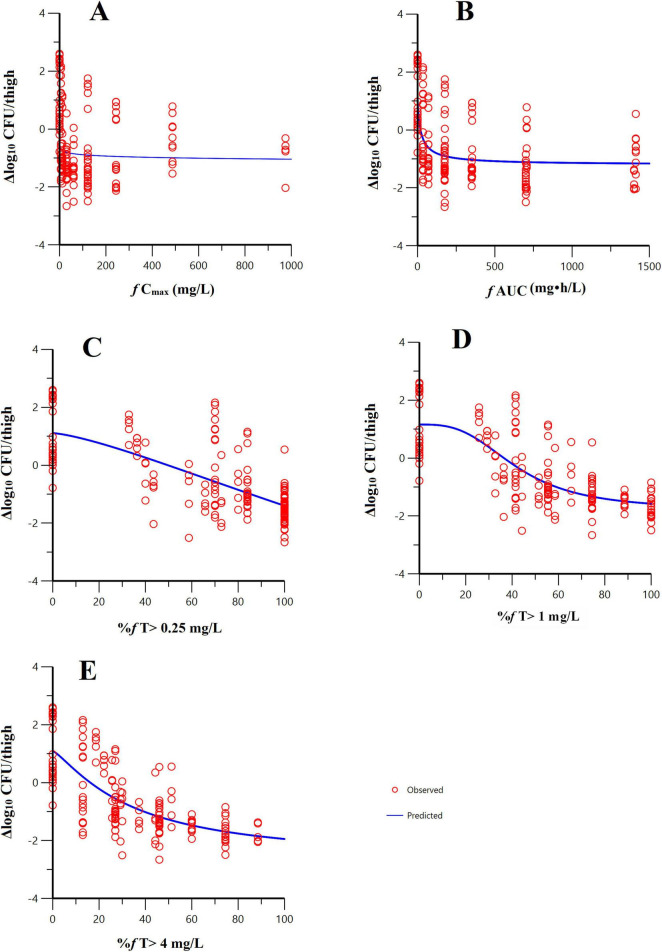
E_max_ model fitting of the pharmacokinetic/pharmacodynamic (PK/PD) index-bactericidal effect of pralurbactam (combined with meropenem). PK/PD index: **(A)** *f* AUC; **(B)** *f* C_max_; **(C)** %*f*T > 0.25 mg/L; **(D)** %*f*T > 1 mg/L; **(E)** %*f*T > 4 mg/L. The hollow circles represent the observed values and the solid lines represent the predicted values. It was evident that the PK/PD relationship of pralurbactam (combined with meropenem) was best described by %*f*T > 1 mg/L.

Sigmoid curves were fitted by regression analysis with PK/PD index expressed as (i) the fraction of the dosing interval above a free threshold concentration (%*f*T > C_T_), (ii) the area under the free drug concentration-time curve (*f*AUC), and (iii) the maximum free drug concentration (*f*C_max_). The fitting results of the correlation between the three PK/PD indexes for pooled *K. pneumoniae* and *E. coli* are shown in Figure 3. The PK/PD relationship of pralurbactam/meropenem was best described by %*f*T > C_T_ (with C_T_ = 1 mg/L) followed by *f*AUC and *f*C_max_. The *R*^2^ is significantly higher than other values (Table 2, 0.733 vs. others). The separate E_max_ model fitting for *K. pneumoniae* and *E. coli* is shown in Supplementary Figure 1, respectively. The E_max_ model fitting for the five strains is shown in Supplementary Figure 2. The estimated values of E_max_ model parameters are shown in Table 2. When the PK/PD index %*f*T > 1 mg/L reached 38.4% and 63.6%, pralurbactam/meropenem combination would achieve bacteriostatic effect and 1-log_10_ reduction against *K. pneumoniae* (KPC and OXA) in thigh bioburden, respectively (Table 3). %*f*T > 1 mg/L for pralurbactam/meropenem combination that achieved a static effect, and 1-log_10_ kill in colony count against *E. coli* (NDM) were 1.8 and 40.1, respectively (Table 3).

**TABLE 2:** E_max_ model estimates and PK/PD targets of pralurbactam (combined with meropenem).

	Strain no.	Type of β -lactamase	PK/PD index	*R*	E_max_	EC_50_	E_0_	γ
Overall	NA	NA	*f* C_max_	0.555	4.12	300	1.10	0.0651
			*f* AUC	0.517	2.33	21.3	1.11	0.884
			%*f*T > 0.25 mg/L	0.699	11.8	262	1.11	1.37
			%*f*T > 1 mg/L	0.733	2.94	42.1	1.16	3.00
			%*f*T > 4 mg/L	0.707	3.83	33.8	1.10	1.25
*Klebsiella pneumoniae*	ATCC BAA-1705	KPC-2	%*f*T > 1 mg/L	0.843	3.37	52.5	0.424	0.219
	17-R1-016	KPC-2	%*f*T > 1 mg/L	0.809	4.23	31.8	2.44	2.59
	20-W2-70	OXA-48	%*f*T > 1 mg/L	0.919	4.14	55.8	2.22	5.68
	All		%*f*T > 1 mg/L	0.754	3.98	43.8	1.72	2.06
*Escherichia coli*	18-W32-020	NDM-5	%*f*T > 1 mg/L	0.946	2.35	32.4	0.287	1.42
	18-W39-088	NDM-1	%*f*T > 1 mg/L	0.861	1.47	41.0	0.135	4.39
	All		%*f*T > 1 mg/L	0.893	3.48	101	0.211	0.680

The analysis was performed only for the strains with significant differences in bactericidal effect. The administration of meropenem alone exhibited significant inhibitory effects on 18-W40-096 or ATCC BAA-2452. AUC, area under the concentration-time curve from 0 to 24 h; C_max_, maximum concentration; E_0_, baseline effect; EC_50_, the concentration of the drug that gives half-maximal response; E_max_, maximum effect; *f*, free drug; NA, not available; PK/PD, pharmacokinetic/pharmacodynamic; γ, Hill coefficient; %*f*T > C_T_, the percent time of free drug above C_T_ (C_T_, threshold concentration: 0.25, 1, and 4 mg/L).

**TABLE 3:** Targets of the pharmacokinetic/pharmacodynamic indexes for pralurbactam (combined with meropenem).

	Strain no.	Type of β -lactamase	PK/PD index	Bacteriostasis	1-Log kill
*Klebsiella pneumoniae*	ATCC BAA-1705	KPC-2	%*f*T > 1 mg/L	NA	12.6
	17-R1-016	KPC-2	%*f*T > 1 mg/L	35.8	56.1
	20-W2-70	OXA-48	%*f*T > 1 mg/L	57.2	69.6
	All		%*f*T > 1 mg/L	38.4	63.6
*Escherichia coli*	18-W32-020	NDM-5	%*f*T > 1 mg/L	8.1	37.1
	18-W39-088	NDM-1	%*f*T > 1 mg/L	24.4	54.1
	All		%*f*T > 1 mg/L	1.8	40.1

The analysis was performed only for the strains with significant differences in bactericidal effect. NA, not available.

## 4 Discussion

In this study, the PK parameters of pralurbactam were calculated for each dosage cohort using Phoenix WinNonlin software based on the mean plasma concentrations at each sampling point in mice. The mean values of the PK parameters were used to simulate the potential PK/PD indices. Another method, the PK parameters could be estimated by the population PK (PPK) model using NONMEM software based on the pooled plasma concentrations. The estimated typical values of K_e_ and V/F based on the population PK model of pralurbactam were generally consistent with the classical mean values of K_e_ (PPK: 2.34 h^1^ vs. classical PK: 2.55 h^1^) and V/F (PPK: 0.719 L vs. classical PK: 0.72 L). K_a_ estimate was close to the median value (PPK: 29.1 h^1^ vs. classical PK: 26.0 h^1^). *K*_a_ could not be obtained accurately because the peak concentration was reached at the first sampling time point (5 min after intraperitoneal injection of pralurbactam). The classical K_a_ varied greatly between dosage cohorts (range from 13.7 to 159.4 h^1^). However, the effect of K_a_ variation on PK/PD index could be negligible, and the inter-mice variation did not have significant effect on the result of PK parameters.

Most drugs are eliminated more rapidly in mice than in human body. Multiple doses are required in mice to simulate the absorption, distribution, metabolism, and excretion process in human body. A dose of 50 mg/kg q2h pralurbactam intraperitoneally injected in mice approximates 500 mg q8h pralurbactam intravenous 2 h-infusion in humans (%*f*T > 1 mg/L: 88% vs. 86%) (Huang et al., 2024b). A dose of 100 mg/kg of meropenem q2h in mice produces a free drug time above 8 mg/L similar to that with 2 g administered q8h by a 2-h infusion in humans, and 50 mg/kg q2h in mice similar to 1 g q8h by a 2-h infusion in humans (Sabet et al., 2018). Monogue et al. (2018) reported that meropenem dosed at 50 mg/kg at 0 h, 8 mg/kg at 2.5 h, and 5 mg/kg at 4.5 h in mice humanized pharmacokinetic profiles compared to human meropenem doses (1 g q8h by a 0.5-h infusion).

*In vitro* studies of pralurbactam proposed %*f*T > C_T_ (C_T_ = 1 mg/L) as the best PK/PD index to predict the *in vivo* bactericidal effect of pralurbactam/meropenem combination (Huang et al., 2024a). The *in vitro* target value was lower because of possible host and bacterial factors (PK/PD 1-log kill target *in vitro*: 48 vs. *in vivo*: 63.6). Much slower growth rate *in vivo* and the influence of the host might lead to this discrepancy (Zhang et al., 2022). Differences in PK profiles between animals and humans require further simulations. Lung infection model is more suitable to investigate the *in vivo* bactericidal activity of pralurbactam/meropenem combination against β-lactamases-producing *K. pneumoniae* because of different drug exposure and the permeability between lung and thigh tissues. It was reported that the PK/PD target obtained in thigh infection model was higher than lung infection model (Berkhout et al., 2016). The thigh model appeared more conservative and stringent values. Further experiments are required to confirm the antimicrobial activity of different pralurbactam/meropenem combinations in mouse lung infection models.

The time above threshold %*f*T > (C_T_ = 1 mg/L) is typically considered the best PK/PD index to predict the bactericidal activity of avibactam, another BLI with a diazabicyclo structure, in the latest studies (Berkhout et al., 2016). However, the conclusion is inconsistent in different animal models and different BLs because AUC may also play a role (MacGowan et al., 2017; Mavridou et al., 2015). The proposal of “time above instantaneous MIC” may explain the differences (Nichols et al., 2022; Bhagunde et al., 2012), but it is impossible to monitor the instantaneous drug concentration in blood in clinical practice. The best PK/PD index of pralurbactam is different from that of other BLIs (Supplementary Table 3; Berkhout et al., 2016; Nichols et al., 2018; Wu et al., 2018; Mavridou et al., 2015; Griffith et al., 2019). The PK/PD target of the same BLI may be different in various infection models such as hollow fiber infection model, thigh infection model, or lung infection model. No clinical PK/PD target values have been reported for pralurbactam. PK/PD study in subsequent clinical trials is also required to refine the exposure-response of pralurbactam/meropenem combinations in humans.

During selection of the lead compounds, pralurbactam showed superior antimicrobial activity than avibactam, which is consistent with previously reported underlying mechanism of resistance to avibactam, including β-lactamase variants, changes in bacterial membrane permeability, and overexpression of efflux pumps (Xiong et al., 2022; Bush and Bradford, 2019), especially the bla_KPC_ gene mutation. PD study demonstrated stronger inhibitory effect of pralurbactam combined with meropenem on KPC-producing bacteria, which corroborates with previous findings (unpublished data from pre-clinical trials). Antimicrobial susceptibility testing results showed that meropenem combined with pralurbactam could not inhibit NDM-producing *K. pneumoniae*, but could inhibit NDM-producing *E. coli* to some extent, which may be attributed to the partial activity of pralurbactam alone against *E. coli* (Huang et al., 2024a). The time-killing studies of different dosing regimens and dose-finding studies in dose-fractionation and dose-escalation experiments are warranted to clarify the most proper dosing regimens in clinical trials.

## 5 Conclusion

In conclusion, %*f*T > 1 mg/L was the PK/PD index that best described the bacterial killing effect of pralurbactam/meropenem over 24 h in mouse thigh infection model. When %*f*T > 1 mg/L reached 38.4% and 63.6%, pralurbactam/meropenem combination would achieve bacteriostatic effect and 1-log_10_ reduction against *K. pneumoniae* in thigh bioburden, respectively. These PK/PD data derived from mouse thigh infection models will be used to inform the optimal dosing regimen of pralurbactam/meropenem combination in clinical trials.

## Data Availability

The raw data supporting the conclusions of this article will be made available by the authors, without undue reservation.
