# Supplementary material for: Pharmacokinetic/pharma-codynamic study of pralurbactam (FL058) combined with meropenem in a neutropenic murine thigh infection model
Source: Front Microbiol. 2024 Dec 17;15:1516979. doi: 10.3389/fmicb.2024.1516979 (PMC11685127; doi:10.3389/fmicb.2024.1516979)
Supplement: Supplementary file 1 [file Data_Sheet_1.docx]

Supplementary Material

**Supplementary Table 1** Susceptibility testing results of meropenem alone, pralurbactam alone, or meropenem with FL058 in combination against *K. pneumoniae* and *E. coli*

|  |  |  |  |  | MIC of meropenem (in combination with pralurbactam) | | | |
| --- | --- | --- | --- | --- | --- | --- | --- | --- |
|  |  | Type of beta-lactamase | Meropenem | FL058 | 1:1 | 2:1 | 4:1 | Meropenem: 4 μg/mL pralurbactam |
| *K. pneumoniae* | ATCC BAA-1705 | KPC-2 | 16 | 4 | 0.5 | 0.5 | 1 | ≤ 0.06 |
|  | 17-R1-16 | KPC-2, CTX-M-14 | >64 | >64 | 2 | 4 | 4 | 0.25 |
|  | 20-W2-70 | OXA-48 | 32 | 8 | 4 | 8 | 8 | 1 |
| *E. coli* | ATCC BAA-2452 | NDM-1 | 32 | 4 | 2 | 4 | 8 | ≤ 0.06 |
|  | 18-W32-020 | NDM-5 | 32 | 2 | 1 | 2 | 4 | ≤ 0.06 |
|  | 18-W39-088 | NDM-1 | >64 | 4 | 2 | 4 | 8 | 0.5 |
|  | 18-W40-096 | NDM-1 | 64 | 4 | 2 | 4 | 8 | ≤ 0.06 |

**Supplementary Table 2** Dosing regimens of pralurbactam in combination with meropenem for pharmacokinetic/pharmacodynamic analysis

| Isolate no. | Pralurbactam | | Meropenem | |
| --- | --- | --- | --- | --- |
|  | Dosing interval | Dosage | Dosing interval | Dosage |
| 17-R1-016 | q2h | 5 mg/kg, 25 mg/kg, 100 mg/kg, 200 mg/kg | q2h | 100 mg/kg |
|  | q4h | 50 mg/kg, 100 mg/kg, 200 mg/kg, 400 mg/kg | q2h | 100 mg/kg |
|  | q8h | 100 mg/kg, 200 mg/kg, 400 mg/kg, 800 mg/kg | q2h | 100 mg/kg |
|  | NA | NA | q2h | 100 mg/kg |
| ATCC BAA-1705  20-W2-70 | q2h | 5 mg/kg, 10 mg/kg, 25 mg/kg, 100 mg/kg | q2h | 100 mg/kg |
|  | NA | NA | q2h | 100 mg/kg |
| ATCC BAA-2452  18-W32-020  18-W39-088  18-W40-096 | q2h | 5 mg/kg, 10 mg/kg, 25 mg/kg, 100 mg/kg | q2h | 50 mg/kg |
|  | NA | NA | q2h | 50 mg/kg |

**Supplementary TABLE 3** PK/PD indexes and targets of novel β-lactams/β-lactamase inhibitors

| **β-lactam/β-lactamase inhibitor** | **β-lactamase** | **Animal model** | **Bacteria** | **PK/PD index** | **Stasis** | **1-log_10_** | **2-log_10_** | **References** |
| --- | --- | --- | --- | --- | --- | --- | --- | --- |
| CAZ/AVI | AmpC | Lung infection in mice | PSA | %*f* T>1mg/L | 20 | 24 | - | 1 |
| CAZ/AVI | AmpC | Neutropenic murine thigh infection | PSA | %*f* T>1mg/L | 40 | 50 | - | 2 |
| IMP/REL | KPC-2/3 | Neutropenic murine thigh infection | KPN, PSA | *f*AUC/MIC | 16 | - | - | 3 |
| IMP/REL | KPC-2/3 | Hollow Fiber | EC, KP, PSA | *f*AUC/MIC_I-R_ | 8 | 12 | 18 | 4 |
| MEM/VAB | KPC | Hollow Fiber | KNP, ENC | *f*AUC/MIC | 12 | 18 | 25 | 5 |
| MEM/VAB | KPC | Neutropenic murine thigh infection | ECO, KPN, ENC | *f*AUC/MIC | 9 | 38 | 220 | 5 |

CAZ/AVI, ceftazidime/avibactam; ATM/AVI, aztreonam/avibactam; IMP/REL, imipenem/relebactam; MEM/VAB, meropenem/vaborbactam; PSA, *Pseudomonas aeruginosa*; ECO, *E. coli*; KPN, *K. pneumoniae*; ENC, *Enterobacter cloacae*; MIC_I-R_, intermediate or resistant.


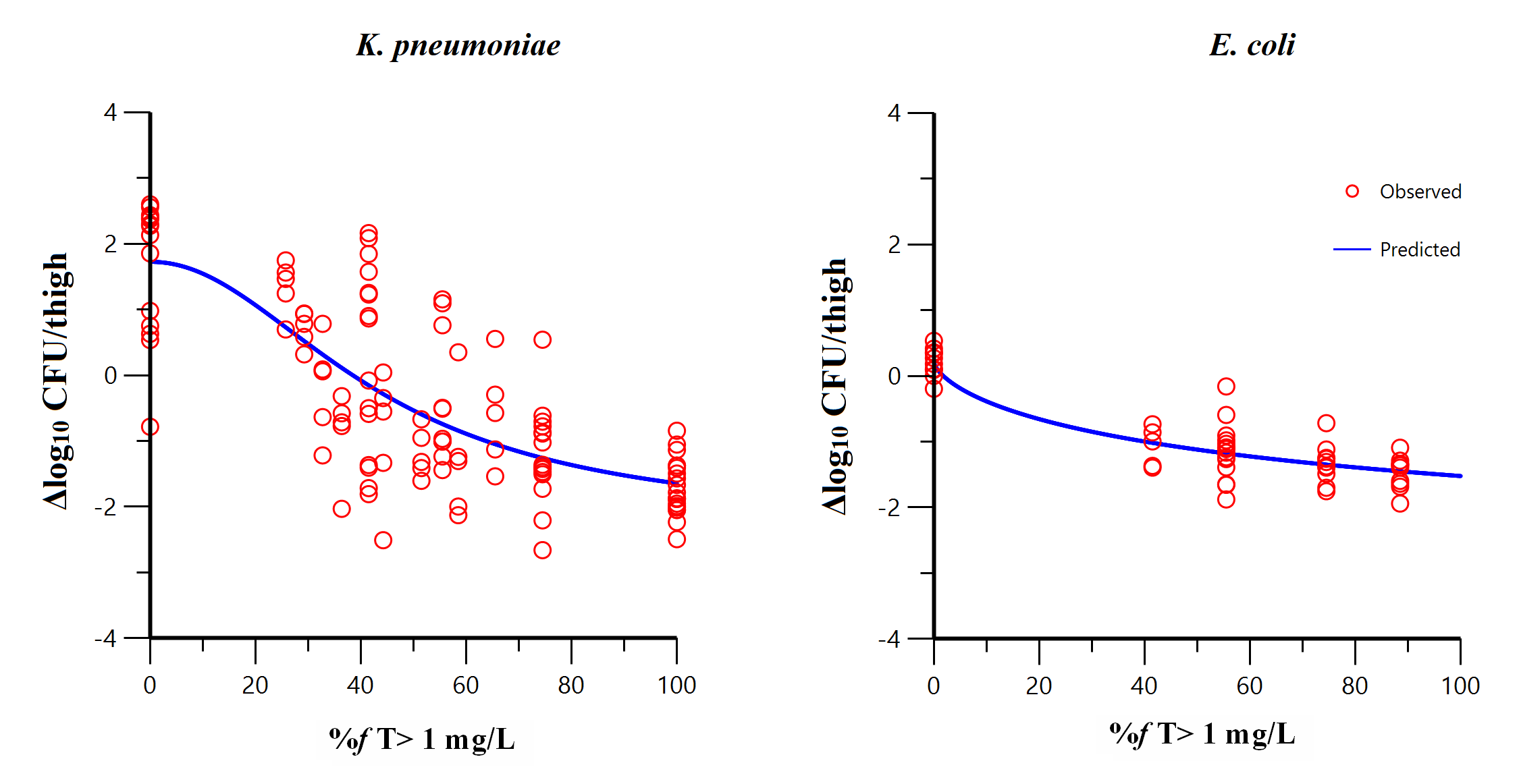


**Supplementary Figure 1** The E_max_ model fitting of the pharmacokinetic/pharmacodynamic index-bactericidal effect of pralurbactam (combined with meropenem) for *K. pneumoniaeare* and *E.coli*


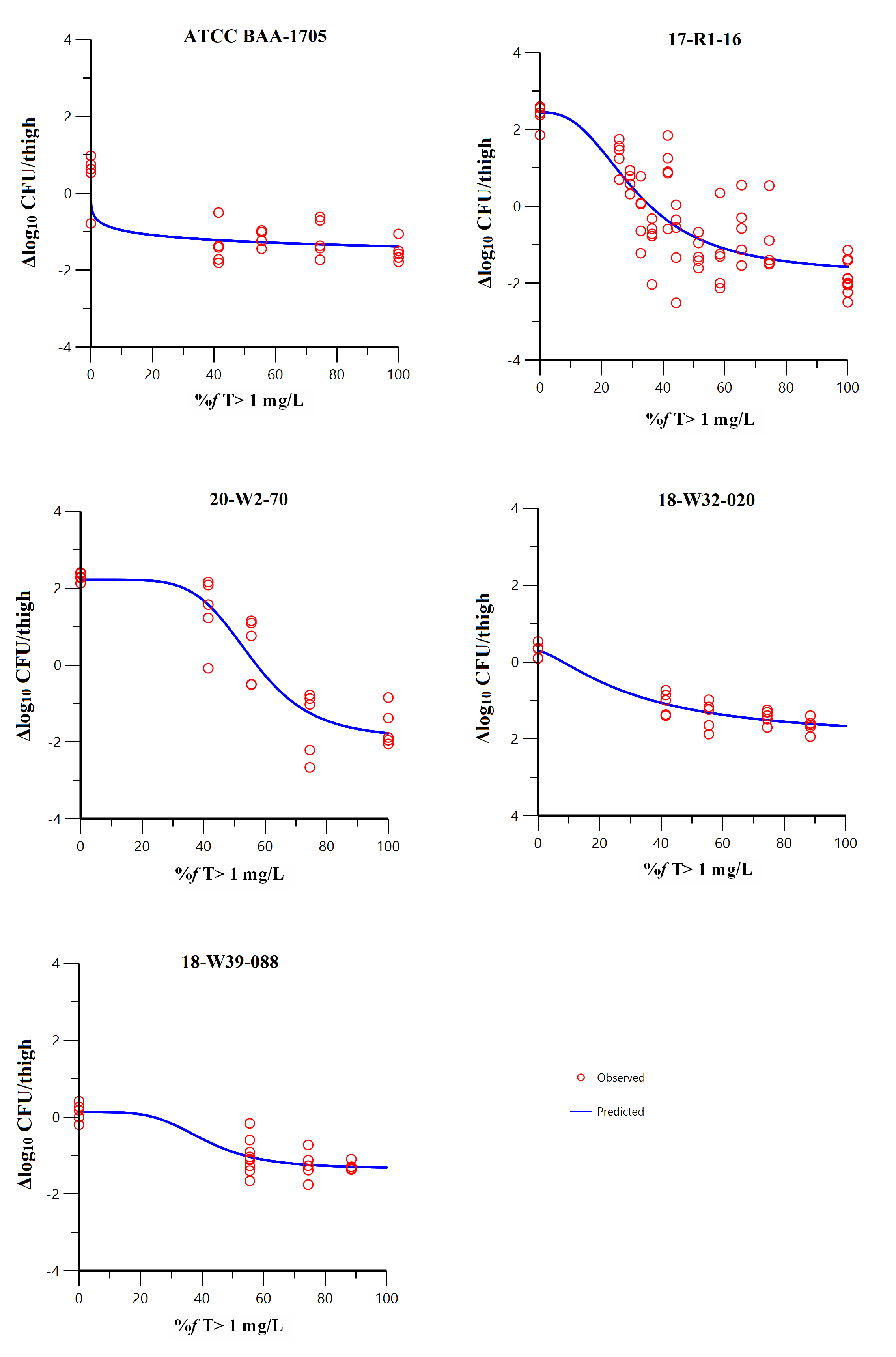


**Supplementary Figure 2** The E_max_ model fitting of the pharmacokinetic/pharmacodynamic index-bactericidal effect of pralurbactam (combined with meropenem) for the five evaluable strains.

1. Berkhout, J., Melchers, M. J., van Mil, A. C., Seyedmousavi, S., Lagarde, C. M., Schuck, V. J., Nichols, W. W., & Mouton, J. W. (2015). Pharmacodynamics of Ceftazidime and Avibactam in Neutropenic Mice with Thigh or Lung Infection. *Antimicrob Agents Chemother*. 60(1), 368–375. <https://doi.org/10.1128/AAC.01269-15>
2. Nichols, W. W., Newell, P., Critchley, I. A., Riccobene, T., & Das, S. (2018). Avibactam Pharmacokinetic/Pharmacodynamic Targets. *Antimicrob Agents Chemother*. 62(6), e02446-17. <https://doi.org/10.1128/AAC.02446-17>
3. Wu, J., Racine, F., Wismer, M. K., Young, K., Carr, D. M., Xiao, J. C., Katwaru, R., Si, Q., Harradine, P., Motyl, M., Bhagunde, P. R., & Rizk, M. L. (2018). Exploring the Pharmacokinetic/Pharmacodynamic Relationship of Relebactam (MK-7655) in Combination with Imipenem in a Hollow-Fiber Infection Model. *Antimicrob Agents Chemother*. 62(5), e02323-17. <https://doi.org/10.1128/AAC.02323-17>
4. Mavridou, E., Melchers, R. J., van Mil, A. C., Mangin, E., Motyl, M. R., & Mouton, J. W. (2015). Pharmacodynamics of imipenem in combination with β-lactamase inhibitor MK7655 in a murine thigh model. *Antimicrob Agents Chemother*. 59(2), 790–795. <https://doi.org/10.1128/AAC.03706-14>
5. Griffith, D. C., Sabet, M., Tarazi, Z., Lomovskaya, O., & Dudley, M. N. (2018). Pharmacokinetics/Pharmacodynamics of Vaborbactam, a Novel Beta-Lactamase Inhibitor, in Combination with Meropenem. *Antimicrob Agents Chemother*. 63(1), e01659-18. <https://doi.org/10.1128/AAC.01659-18>
